# Age related Antibiotic Prescribing Trends of Clostridioides Difficile Incident Cases within Davidson County TN 2012-2020

**DOI:** 10.1017/ash.2024.181

**Published:** 2024-09-16

**Authors:** Michael Norris, Priscilla Pineda, Malakai Miller, Raquel Villegas

**Affiliations:** State of Tennessee Department of Health; Tennessee Department of Health; State of Tennessee Department of Health State of TN

## Abstract

Age related Antibiotic Prescribing Trends of Clostridioides Difficile Incident Cases within Davidson County Tennessee 2012-2020 Michael Norris, MSN, Priscilla Pineda, MPH, Malakai Miller, MPH, Raquel Villegas, PhD, MS **Background:** Clostridioides difficile infection (CDI) is one of the most common healthcare-associated infections in the United States. Antibiotic use is considered a predisposing factor for CDI. The State of Tennessee collaborates with the CDC as part of an ongoing Emerging Infections Program (EIP). We sought to better understand the impact of antimicrobial use prior to the date of incident of CDI within the defined age groups of Davidson County, Tennessee. **Methods:** Surveillance data from the years 2012–2020 were examined for all positive CDI cases within Davidson County. A positive CDI case was defined as a laboratory confirmed case who is ≥ 1 year old living in Davidson County, Tennessee. Antibiotic use was assessed in the 12 weeks prior to CDI. Trends of overall antibiotic use, including the top five antibiotics prescribed by our defined age groups were examined. Analyses were performed using SAS version 9.4. Only fully abstracted cases are included in the study. **Results:** Among 7,346 positive CDI incident cases identified between 2012–2020, 5,467 (74.4%) received antibiotics 12 weeks prior to a confirmed infection. We looked at the trend of antibiotic prescription over time from 2012-2020 (77.0%, 76.7%, 74.3%, 80.7%, 76.3%, 75.1%, 73.7%, 74.8% and 71.5%) which has decreased since 2015. The prevalence of antibiotic use by age group 1-18 years, 19-44 years, 45-64 years, 65-74 years, and 75+ years was 53.4%, 68.8%, 74.5%, 79.2% and 83.1% respectively. The five most prescribed antibiotics were ceftriaxone ((11.1%), followed by vancomycin IV (10.9%), ciprofloxacin (10.2%), metronidazole (9.1%), and piperacillin (8.6%). Cases in the 45-64 years age group were more likely to be prescribed vancomycin IV, ciprofloxacin, metronidazole, and piperacillin-tazobactam compared to other age groups (p < 0.0001). There was no statistically significant association between ceftriaxone prescription and our defined age groups. **Conclusion:** In this study, almost three quarters of the CDI cases had received antimicrobial therapy in the 12 weeks prior to infection. Since antibiotic prescription is a potentially modifiable risk factor for CDI, a more in-depth study, combined with an antibiotic stewardship program implementation in all settings would be beneficial to reduce the risk of CDI complications of antibiotic usage.

